# Uterine Fibroids: Pathogenesis and Interactions with Endometrium and Endomyometrial Junction

**DOI:** 10.1155/2013/173184

**Published:** 2013-09-12

**Authors:** Andrea Ciavattini, Jacopo Di Giuseppe, Piergiorgio Stortoni, Nina Montik, Stefano R. Giannubilo, Pietro Litta, Md. Soriful Islam, Andrea L. Tranquilli, Fernando M. Reis, Pasquapina Ciarmela

**Affiliations:** ^1^Woman's Health Sciences Department, Faculty of Medicine, Polytechnic University of Marche, Via Corridoni 11, 60123 Ancona, Italy; ^2^Department of Gynaecological Sciences and Human Reproduction, University of Padova, Via Giustiniani 3, 35128 Padova, Italy; ^3^Department of Experimental and Clinical Medicine, Faculty of Medicine, Polytechnic University of Marche, Via Tronto 10/a, 60126 Ancona, Italy; ^4^Department of Obstetrics and Gynecology, Federal University of Minas Gerais and National Institute of Hormones and Women's Health, 30130-100 Belo Horizonte, MG, Brazil

## Abstract

Uterine leiomyomas (fibroids or myomas) are benign tumors of uterus and clinically apparent in a large part of reproductive aged women. Clinically, they present with a variety of symptoms: excessive menstrual bleeding, dysmenorrhoea and intermenstrual bleeding, chronic pelvic pain, and pressure symptoms such as a sensation of bloatedness, increased urinary frequency, and bowel disturbance. In addition, they may compromise reproductive functions, possibly contributing to subfertility, early pregnancy loss, and later pregnancy complications. Despite the prevalence of this condition, myoma research is underfunded compared to other nonmalignant diseases. To date, several pathogenetic factors such as genetics, microRNA, steroids, growth factors, cytokines, chemokines, and extracellular matrix components have been implicated in the development and growth of leiomyoma. This paper summarizes the available literature regarding the ultimate relative knowledge on pathogenesis of uterine fibroids and their interactions with endometrium and subendometrial myometrium.

## 1. Introduction

Leiomyomas are benign uterine tumors of unknown aetiology. These kinds of lesions seem to arise from myometrial transformation as a result of specific physiological and pathological conditions. The majority of these monoclonal estrogen-dependent uterine neoformations [1] afflict mostly women during reproductive age, and 80% of them suffer from this during their whole lifetime [2]. In the past, most women with fibroids remained undiagnosed, because they were asymptomatic. Analyses based on clinical diagnosis or diagnostic tests underestimate the true incidence; in fact, they take only into account symptomatic patients. 

Cramer and Patel [3] estimated the prevalence of uterine fibroids based on clinical assessment at 33%, ultrasound scan at 50%, and histological examination of hysterectomy specimens at 77%. The reported frequency of the disease varies widely due to differences in study design. In fact, to determine the exact prevalence of fibroids, a correct clinical research should apply ultrasound scanning in a randomly sampled population [4].

Nowadays, conflicting data about the pathogenesis of leiomyomas coexist in the literature. The development of uterine myomas can be linked to predisposing risk factors, initiators and genetic mechanisms, promoters, and effectors. The aim of this work is to discuss the latest knowledge on the pathogenesis of uterine fibroids and their interactions with the endometrium and subendometrial myometrium.

## 2. Pathogenesis of Uterine Leiomyoma

### 2.1. Risk Factors

Even if many risk factors suggested by epidemiologic studies have linked uterine leiomyomas to the effects of estrogens and progesterone levels and their metabolism, other mechanisms may be involved in fibroids pathogenesis. In fact, recently, Peddada et al. [5] have questioned the exact role of female hormones (estrogens and progesterone) in the development and growth of uterine fibroids. The authors measured the growth of fibroids in black and white women with clinically relevant fibroids using MRI technology; they demonstrated that fibroids within the same woman often have different growth rates despite having a similar hormonal milieu. In the same patients, fibroids were found to vary in size, regress, or remain stable. Each tumor appeared to have its own intrinsic growth rate, and fibroid growth appeared not to be influenced by tumor characteristics such as size and location. This study encouraged new research directions, consistent with studies showing that fibroids are monoclonal in origin with variable molecular characteristics [6–9]. Wei et al. [7] also found ethnic differences in expression of the dysregulated proteins in uterine leiomyomata. Tumor size has been related to variation in molecular markers [6–8, 10], and it has been assumed that the molecular differences reflect differences in tumor growth rates. Moreover, molecular markers also may differ between tumors from blacks and whites [7, 10]. It has been generally accepted that myomas are more prevalent in blacks than in Caucasian and Hispanic populations [11, 12]. Although the cause of the higher prevalence among black women is unclear, differences in circulating estrogen levels have been found [13]. It is still unclear [14] whether these ethnical differences are genetic or due to known variations in hormonal metabolism, diet, or environmental factors. Recently, some authors reported a statistically significant inverse correlation between serum 25-(OH) Vit D levels and fibroid prevalence in black subjects [15, 16]. Leppert et al. reported that the pathogenesis of fibroids seems to involve a positive feedback loop between extracellular matrix production and cell proliferation, and vitamin D might act to block the positive feedback [17]. It is also interesting that myomas and keloids, both more common in black women, have similar gene characteristics. Furthermore, it is well known that family history could represent a strong predisposing factor; the first-degree relatives of affected women have a 2.5 times increased risk of developing fibroids [18, 19]. However, as recently reported from Saldana et al., such bias would invalidate self-reported family history as a predictor of fibroid risk [20]. 

Several studies [13, 14, 18, 19] reported a rapid increase of fibroid incidence after the age of 30. This could be the result of time-related hormonal changes or an enhanced symptomatology from already existing fibroids. Furthermore, the high incidence of fibroids in the perimenopausal period could be responsible for increasing gynecologic surgery rates in women who have completed the childbearing period.

A study found that the risk of myomas increased 21% with each 10 kg increase in body weight and with increasing body mass index [21]. Shikora et al. reported similar results in women with greater than 30% body fat [22]. The adipose tissue converts adrenal and ovarian androgens into estrogens, whereas several mechanisms associated with obesity lead to decreased synthesis of sex hormone binding globulin. Consequently, the increase of biologically available estrogens could be responsible for increasing myoma prevalence and/or growth in overweight and obese women. Furthermore, Nair and Al-Hendy evaluated the association between obesity-related chronic inflammation and initiation, as well as the progression of uterine leiomyoma by using an *in vitro* model with representative cell lines of adipocytes and human uterine leiomyoma cells. They demonstrated that coculture of adipocytes and uterine leiomyoma cells results in an increased proliferation of leiomyoma cells, and they have also demonstrated that TNF-*α* treatment increases human uterine leiomyoma cells proliferation in a concentration-dependent manner [23].

It is not clear whether diet habits, such as consuming red meat, ham, green vegetables, or fiber, could influence the growth of myomas. It is also difficult to analyze the specific effects of physical exercise on the development of uterine myomas, as only a few observational studies have addressed this aspect so far [24, 25]. 

Several studies have revealed that smoking may reduce the incidence of myomas; nicotine inhibits aromatase and reduces the conversion of androgens to estrone. Smoking also exerts a powerful inducing effect on the 2-hydroxylation pathway of estradiol metabolism, which is likely to lead to decreased bioavailability at estrogen target tissues [26–28]. 

An early menarche, before the age of 10, has been found to be a risk factor for uterine myomas, while a menarche over the age of 16 seems to decrease the same risk [29]. Some studies stressed that a lower incidence and a reduced number of clinically apparent myomas are linked to increased parity [30–32]. This could be due to a remodeling process of the extracellular matrix (ECM) and a specific expression of receptors for peptide and steroids hormones induced by pregnancy and parturition. 

Postmenopausal hormone therapy seems not to be responsible for any important stimulus to fibroid growth [33]. Likewise, conflicting data coexist about the relationship between oral contraceptives (OC) and the growth of leiomyomas. This could be related to the differing content of estrogens and the type of progesterone in each specific OC preparation [34]. 

Several theories about the initiators of fibroids have been proposed. Rein [35] stated that increased levels of estrogens and progesterone could result in an augmentation of mitotic rate that could be responsible for somatic mutation. Richards and Tiltman found increased concentration of receptors for estrogens (ER) in certain regions of the nonneoplastic myometrium of uterus myomatous [36]. Another interesting theory underlines that the pathogenesis might be similar to a response to injury [37]; ischemic damage could be linked to release of increased vasoconstrictive substances at the time of the menses. Smooth muscle cells of the myometrium could react to injury with the synthesis of extracellular fibrous matrix [38]. After vascular damage, basic fibroblast growth factors are overexpressed in leiomyomas [39, 40].

### 2.2. Genetic Mechanism Involved in Fibroids Etiology

Historically, uterine leiomyomata have not been considered a genetic disease. However much recent clinical evidence indicates that at least some myomata have a genetic etiology. Actually, cytogenetic surveys have found that about 40% of uterine fibroids are chromosomally altered and bear cytogenetic anomalies shared by several other types of tumors. For example, studies found translocations between chromosomes 12 and 14, trisomy 12, translocations between chromosomes 6 and 10, and deletions of chromosomes 3 and 7 [41]. 

The HMGA2 gene was found in translocation 12:14, the most common cytogenetic abnormality, that occurs in about 20% of chromosomally abnormal lesions. This gene encodes a high mobility group DNA binding protein and embryonic proliferation modulator [42]. The HMGA2 gene is expressed in uterine leiomyoma and in other human tissues with a proliferative phenotype, such as fetal tissues, lung, and kidney, but not in the normal myometrium [43]. Markowski et al. found that the antagonism of HMGA2 *in vitro* decreased leiomyoma cell proliferation [44]. 

Heritable cancer syndromes can be characterized by uterine leiomyomas such as hereditary leiomyomatosis and renal cell cancer (HLRCC). This syndrome predisposes patients to benign leiomyomas of skin and uterus and early-onset renal cell carcinoma. Fumarate hydratase (FH) is the gene implied; it encodes a Kreb's cycle enzyme responsible for conversion of fumarate to malate [45]. Alport syndrome is an X-linked progressive nephropathy associated with leiomyomas due to defect in COL4A5 and COL4A6 genes [46].

Cha and colleagues [47] genotyped 1607 individuals with uterine fibroids and identified 3 susceptibility loci associated with uterine fibroids. Chromosome 10q24.33 seems to have the best association with leiomyomas; the region was mapped to the 5′ region of the SLK gene encoding STE20-like kinase. STE20-like kinase has a role in myogenic differentiation, and, after activation by epithelial disruption, it is expressed in proliferating myoblasts. Another gene product located in the region is A-kinase anchor protein-13 (AKAP13), associated with cytoskeletal filaments. Related mutations could alter the regulation of extracellular matrix deposition and, consequently, of the fibrotic phenotype of the leiomyoma [48]. 

Recent studies described that 70% of fibroids contained a series of mutations in a transcriptional regulator complex subunit 12 (MED12) [49, 50]. Pérot et al. reported that MED12 is frequently mutated in typical leiomyomas (66.6%) and also that mutations are not restricted to benign tumors since highly aggressive leiomyosarcomas were also mutated. However, no mutations were detected in nonuterine leiomyosarcomas; so Pérot et al. affirmed that MED12 seems to be specific to uterine smooth muscle tumors [51]. Previously, it has been shown that MED12 is implicated in transcription activation of Wnt target genes by interacting with *β*-catenin [52, 53]. However a recent study combining mRNA and miRNA differential expression between fibroids and myometrium has observed a downregulation of the Wnt pathway and an upregulation of the focal adhesion pathway in leiomyomas [54]. The *β*-catenin immunohistochemistry data tends to indicate that the canonical Wnt pathway is not implicated in fibroids development, since *β*-catenin, when expressed, is located at the membrane in mutated cases; a localization which has been demonstrated to be indicative of a low transactivation activity [51, 55, 56].

The same authors [51] concluded that the Wnt/*β*-catenin pathway does not seem constitutively activated in MED12 mutated tumors, and they hypothesize that if MED12 mutations play a role in uterine tumor development, it is probably not through Wnt target genes activation in association with *β*-catenin.

### 2.3. Role of Mechanical Transduction and Extracellular Matrix

Research for the pathogenesis of fibroids and abnormal extracellular matrix (ECM) led to the analysis of a growth factor with profibrotic activity, transforming growth factor *β* (TGF-*β*) [17, 57]. The *β*3 subunit of TGF-*β*3 and its signal mediators are overexpressed in leiomyomas compared to normal myometrium [58]. Furthermore, the mRNA expression of multiple ECM genes in uterine leiomyomas is decreased when the TGF-*β* pathway is downregulated [59].

Norian et al. have examined the role of ECM, opening new directions of research. They reported that mechanical signals are transmitted from the ECM scaffold via transmembrane receptors to the internal cytoskeleton in order to maintain an isometric state. Transmembrane receptors respond to stretch, fluid shear stress, elevated hydrostatic pressure, and increased osmotic forces. In this way, myometrial cells react to, and may be protected from, external loads by the mechanical properties of the surrounding matrix through secretion of ECM. The authors [60] have demonstrated that the ECM microenvironment of leiomyoma cells is characterized by increased mechanical stress. They extended the results of their previous study [48] showing that the viscoelastic properties of the ECM contribute substantially to the increased tissue stiffness of leiomyoma. They hypothesized that since the viscoelastic properties of the ECM are complex, it is possible that the interstitial fluid may alter the repulsive forces of the glycosaminoglycans allowing them to collapse or inflate. So the authors [60] suggested that the mechanical properties of leiomyoma are a key feature of these tumors and may contribute to their growth.

### 2.4. MicroRNA

Epigenetic changes have also been implicated in leiomyoma formation. Studies directed at identifying epigenetic abnormalities in fibroids demonstrated abnormally hypomethylated ER-*α* [61]. Follow-up studies demonstrated globally abnormal genomic methylation in leiomyomas compared to myometrium [62], implicating possible epigenetic contributions to genetic susceptibility of leiomyoma development. Knowledge regarding the molecular causes of uterine leiomyomas is in its infancy. Early studies suggest common mutations that correlate with the development of leiomyomas. MicroRNAs (miRNAs) are a novel class of small nonprotein coding RNAs which regulate a high number of biological processes by targeting mRNAs for cleavage or translational repression [63, 64]. Several miRNAs such as let7, miR-21, miR-93, miR-106b, and miR-200 are significantly dysregulated in uterine leiomyoma compared to those in normal myometrium [10, 65]. Further research will need to identify specific genes responsible for the development of leiomyomas that can be directly targeted as preventive therapy. Additional efforts need to be directed at investigating specific inhibitors of disrupted pathways involved in the leiomyoma growth in susceptible patients. The wide spectrum of clinical and genetic heterogeneity of uterine leiomyomas underscores the importance of continued investigation to determine the various molecular etiologies that result in leiomyoma development. 

### 2.5. Estrogens

Uterine leiomyoma growth is strictly related to estrogens and their receptors. Several studies found that mRNA and protein expression levels as well as the content of ER-*α* and ER-*β* are higher in leiomyoma compared to those in normal myometrium [66, 67]. According to their hypothesis, estrogens may exert their growth-stimulatory effects on leiomyomas intermediated by cytokines, growth factors, or apoptosis factors [68]. Ishikawa et al. [69] suggested that estrogens can maintain progesterone receptor (PR) levels, and thus progesterone through its receptor may promote leiomyoma growth. Furthermore, other authors suggested that estrogens may stimulate leiomyoma growth partially by suppressing normal p53 functions [70].

Estrogens are able to regulate the expression of growth factors by activating some signaling pathways. Estrogens upregulate platelet-derived growth factor (PDGF) expression [71] in leiomyoma cells, while they downregulate activin and myostatin [72] in human myometrial explants. In addition, estrogens also downregulate epidermal growth factor (EGF) expression but upregulate the expression of EGF-R in both myometrium and leiomyoma cells [73, 74]. These estrogen actions are accomplished through the rapid activation of different kinds of kinases; some of them [75] result to be increased in both immortalized uterine smooth muscle and leiomyoma cell lines under estrogen stimulation. In addition, Park and colleagues reported that estrogens may also stimulate the proliferation of leiomyoma cells by activating ATP-sensitive potassium channels [76].

### 2.6. Progesterone

Progesterone interacts with its receptors PR-A and PR-B [77] playing a key role in myometrial and leiomyoma biologies [78, 79]. Several studies have stressed that PR content and mRNA levels are higher in leiomyoma than those in normal myometrium [80–84], and, in particular, Fujimoto et al. [85] described the relative overexpression of PR-B mRNA in the surface of leiomyoma.

Leiomyoma growth is influenced by progesterone interaction with some growth factors; it upregulates the EGF (mitogenic) [73] and transforming growth factor- (TGF-)*β*3 (bimodal action) [86] expression. On one hand, progesterone seems to downregulate IGF-I expression through PRB, while PRA appears to inhibit this function [84].

Some authors hypothesized that progesterone could stimulate leiomyoma cell growth and survival through upregulating B-cell lymphoma- (Bcl-)2 protein expression and downregulating tumour necrosis factor- (TNF-)*α* expression [87, 88]. Recently, Luo et al. [89] defined L-type amino acid transporter 2 (LAT2) as a novel PR target gene. Progesterone significantly induces LAT2 mRNA levels, which is blocked by cotreatment with the PR antagonist mifepristone. In the same way, Yin et al. found eighteen novel PR-binding sites, one of which is Krüppel-like transcription factor 11 (KLF11) which is minimally downregulated by progesterone [90].

### 2.7. Growth Factors

Several growth factors, such as vascular endothelial growth factor (VEGF), EGF, heparin binding epidermal growth factor (HB-EGF), PDGF, IGF, TGF-*α*, TGF-*β*, acidic fibroblast growth factor (aFGF), and basic fibroblast growth factor (bFGF), and their respective receptors have been demonstrated to play a role in leiomyoma growth [91, 92]. In particular, bFGF [93] and VEGF [94] have also been shown to promote angiogenesis in leiomyoma. EGF and PDGF seem to increase DNA synthesis and polyploidization in leiomyoma cells through transient activation of kinase pathways [95–97]. PDGF also modulates the rate of cell proliferation in myometrium and leiomyoma cells [98–100].

TGF-*β*3 induces elevated expression of ECM-related genes and decreases the expression of ECM degradation-related genes [101]. TGF-*β* can also activate kinase pathways (MAPK/ERK/Smad) and thereby modulate the expression of different types of genes influencing the leiomyoma growth and regression [102]. Similarly, IGF may increase cellular proliferation in uterine leiomyoma cells through activation of the MAPK pathway [103] and thus play a crucial role in leiomyoma cell growth, by upregulation of Bcl-2 protein expression in leiomyoma cells [104].

Recently, activin and myostatin have been identified in the myometrium and in leiomyoma, and Ciarmela et al. [105, 106] have hypothesized that activin-A and myostatin could regulate myometrial cell proliferation, describing higher expression levels of this molecule in leiomyoma compared to that in adjacent myometrium samples. Additionally, several less studied factors such as parathyroid hormone-related peptide [107, 108], prolactin [109, 110], endothelin-1 [111, 112], human chorionic gonadotropin [113], and pituitary tumor-transforming growth factor-1 [114] have also been implicated or hypothesized in myometrial biology.

### 2.8. Cytokines and Chemokines

Many cytokines, including tumor necrosis factor-*α* [87], erythropoietin [115], interleukin- (IL-)1 [116], and IL-6 [117], have been implicated in development of uterine leiomyoma. Even chemokines and their receptors (MIP-1*α*, MIP-1*β*, RANTES, eotaxin, eotaxin-2, IL-8, CCR1, CCR3, CCR5, CXCR1, and CXCR2 mRNA) have been shown to be mediators of the above mentioned process [118–120]. Sozen et al. [121] found that MCP-1 mRNA levels are higher in myometrium compared to leiomyoma and that estrogens and progestins decrease MCP-1 protein production, suggesting that MCP-1 may have antineoplastic activity in leiomyoma. IL-8 and IL-8 receptors type A have been identified with the elevated expression in myometrium compared to leiomyoma [122]. Hatthachote and Gillespie [116] described that this chemokine also upregulates TGF-*β*1 and TGF receptor expression *in vitro* in human term myometrium.

In experimental systems, increasing carcinogen exposure tends to increase the number of tumors and their degree of malignancy. Low carcinogen exposure tends to produce benign neoplasms, whereas high exposure tends to produce both malignancies and higher numbers of tumors [3, 123, 124]. 

### 2.9. Extracellular Matrix Components

Extracellular disorganized matrix is a peculiar characteristic of fibroid growth, mainly consisting of collagen subtypes, fibronectin, and proteoglycans. Recently, a series of collagen subtypes, such as COL1A1, 4A2, 6A1, 6A2, 7A1, and 16A1, have been found expressed to a greater extent in leiomyoma cells compared to myometrial cells [125]. Leiomyomas and myometrium are characterized by variable expression of glycosaminoglycans and their protein bound forms, proteoglycans [126, 127]. Matrix metalloproteinases (MMPs) have also been implicated in leiomyoma remodeling. Bodner-Adler et al. [128] found that MMP-1 is expressed more in leiomyomas, while MMP-2 is less expressed. Alternatively, another work found MMP-1, MMP-2, MMP-3, and MMP-9 with higher activity of MMP-2 in leiomyoma compared to myometrium [126]. Recently, Bogusiewicz et al. revealed increased MMP-2 activity in leiomyomas than in surrounding myometrium [129]. 

## 3. Fibromatosis, Endometrium, and**** Endomyometrial Junction

The advances in pathogenetic knowledge of fibroids and the introduction of magnetic resonance imaging led to the study of endomyometrial junction [130], the interface between cyclic endometrium and the myometrium, where important vascular and physiochemical phenomena seem to take place. Tocci et al. [131] proposed that the “endometrial-subendometrial myometrium unit disruption disease” should be considered as a new entity and distinguished from adenomyosis. This condition is expressed mainly by a pathological thickening or abnormality of the subendometrial myometrium, that is, the possible site of origin of submucosal and intramural fibroids. The study also reports on the influence of abnormal thickening or disruption on human fertility and outcome of assisted reproduction techniques. The mechanism underlying zonal myometrial differentiation is not known, but growing evidence suggests that ovarian hormone action may be mediated by cytokines and uterotonins locally released by the basal endometrial layer and endometrial-myometrial T-lymphocytes. Irregular *thickening* of the junctional zone due to inordinate proliferation of the inner myometrium, junctional zone hyperplasia, is a common MR finding in women suffering from menstrual dysfunction [132].

Nowadays, it is not well established how uterine fibroids could interfere with the endometrial environment and the subendometrial myometrium and vice versa. In these patients, the interaction between the endomyometrium and fibromatosis could have a role in influencing their fertility and the risk of miscarriage. The American Society for Reproductive Medicine has reported that uterine myomas are associated with infertility in 5–10% of cases [133] and may be responsible for 2-3% of infertility cases [134]. All confounding variables may be difficult to control when searching for the impact of fibroids on infertility, and there is no definitive association between reproductive dysfunction, miscarriage and fibroids. 

Some studies [135–138] indicated that the uterine tissue is susceptible to fibrinogenesis, as seen in rare occasions in response to mechanical injury in women undergoing endometrial ablation and caesarean delivery and in women affected by Asherman's syndrome. In all these cases, in fact, there is an involvement of the subendometrial myometrium, an alteration of endometrial microvascular blood flow with endometrial atrophy and abnormal activation of cytokines and chemokines, which might have an important role in the pathogenesis of uterine leiomyomas and related symptoms. Frequent mucosal injury with stromal repair reactions may release growth factors that promote the high frequency and multiplicity of uterine leiomyomas [139].

A threefold increase in percentage of nuclear area was found in the junctional zone in comparison with the outer myometrium, reflecting an increase in both size and number of nuclei. No difference in distribution of common components of the extracellular space (collagen, laminin, and fibronectin) was found between the two layers [140]. 

It has been reported that myomectomy can increase the pregnancy rate for patients with infertility [141]. However, the mechanisms by which this occurs are not well understood. Many hypotheses have been suggested. First, fibroids could alter uterine cavity contour, through a mechanical distortion, or they could be responsible for an abnormal uterine contractility [142, 143]. Moreover, local inflammation associated with the presence of fibroids may give rise to a hostile endometrial environment that impairs sperm transport and embryo implantation. Some authors [144, 145] reported that excessive concentrations of inflammatory cytokines could have negative effects on embryonic development and implantation. Inagaki et al. [146] demonstrated that uterine cavities containing fibroids or adenomyosis showed a state of excess inflammation, with upregulation of MMPs and inflammatory cytokines such as interleukin-1 and TNF-*α*. In particular, the levels of MMPs in the uterine cavity of women with leiomyoma and adenomyosis were significantly higher than those in women with a histologically normal uterus. Matsuzaki et al. [147] detected significantly lower expression levels of HOXA-10 in patients with uterine leiomyoma. HOXA-10 is one of the best-recognized sequences of signaling events in implantation [148]. 

A study reported that HOXA10 and HOXA11 mRNA expression were significantly decreased in uteri with submucosals myomas compared to those in controls with normal uterine cavity and to uteri with intramural myomas [149]. Although intramural myomas were not associated with a significant change in these markers of endometrial receptivity, the same authors noted a trend toward decreased endometrial HOXA10 mRNA and stromal protein expression in the intramural myoma group compared to those in the control group. Sinclair et al. [150] evaluated the effect of leiomyoma on endometrial gene expression essential for implantation and haemostasis both *in vivo* and in primary endometrial stromal cells. They hypothesized that failure of blastocyst implantation in women with uterine leiomyomas is secondary to impaired BMP-2-mediated decidualization. BMP-2 is a growth factor that belongs to the TGF-*β* superfamily. It regulates cell proliferation and differentiation. Conditional ablation of BMP-2 in murine endometrium results in complete infertility because BMP-2^d/d^ mice are unable to form implantation sites and demonstrate a complete lack of decidual response [151].

The authors tested the hypothesis that TGF-*β*3 may cause impaired decidualization in endometrial stromal cells by inducing BMP-2 resistance. They found a significant reduction in HOXA10 in leiomyoma-associated endometrial stromal treated with rhTGF-*β*3 supporting this hypothesis. TGF-*β*3 downregulated BMP receptors. The significant reduction in BMPR gene expression in response to treatment with rhTGF-*β*3 suggests that TGF-*β*3 induces BMP-2 resistance by downregulation of the BMPRs. Persistently decreased receptor expression explains the continued lack of BMP-2 response of endometrial stromal cells in culture.

Morosova et al. [152] studied common polymorphisms of MMP genes in myometrial and endometrial hyperplasia. An accelerated leiomyoma growth correlated with higher frequency of the MMP-1 2G allele. MMP-1 2G was also associated with multinodular growth, and it also tended to increase in patients with adenomyosis, suggesting that the 2G (-1607)MMP-1 genotype may be a potential risk marker of myometrial and endometrial hyperplasia. 

## 4. Conclusions

The etiopathology of uterine fibroid remains unclear, multifactorial, and enigmatic. Classic studies showed steroid dependence of myomas for growth and development. The genetic background seems to play an important role, with cytogenetic anomalies observed in about 40% of uterine fibroids. Abnormal ECM expression, increased growth factors, cytokines and chemokines concentrations, and an extracellular disorganized matrix have been implicated in development and growth of uterine leiomyomas. 

However, clinical aspects are related prevalently to the number, volume, and intrauterine localization of nodes. In particular, submucosal nodes are associated with important clinical manifestations, and it is still not well established how uterine fibroids could interfere with the endometrial environment and the subendometrial myometrium and vice versa. It has been proposed that the “endometrial-subendometrial myometrium unit disruption disease” should be considered as a new entity and distinguished from adenomyosis [131]. This condition is expressed by a pathological thickening or abnormality of the subendometrial myometrium, that is, the possible site of origin of submucosal and intramural fibroids. Recent findings suggested that myomatosis and adenomyosis share some pathogenetic features like a state of excess inflammation, increased endothelial nitric oxide synthesis with upregulation of MMP, and inflammatory cytokines such as interleukin-1 and TNF-*α*.

Endomyometrial junction disruption might play a crucial role on fibroid-related infertility, uterine bleeding, and growth of submucosal and intramural myomas.

Nowadays, there is a clear trend to delay the time of pregnancy, and the clinical and the social impact of uterine fibromatosis is growing and requires future studies to clarify the etiopathogenesis and elaborate new and effective therapies for this condition.
